# E2F3 accelerates the stemness of colon cancer cells by activating the STAT3 pathway

**DOI:** 10.3389/fonc.2023.1203712

**Published:** 2023-06-29

**Authors:** Qingkun Gao, Ke An, Zhe Lv, Yanzhao Wang, Changmin Ding, Wensheng Huang

**Affiliations:** Department of Gastrointestinal Surgery, Peking University Shougang Hospital, Beijing, China

**Keywords:** E2F3, stemness, STAT3 pathway, colon cancer, cancer

## Abstract

**Introduction:**

Colon cancer is one of the most prevalent malignancies and causes of cancer-related deaths worldwide. Thus, further research is required to explicate the latent molecular mechanisms and look for novel biomarkers. E2F3 has been confirmed to be an oncogene in a variety of cancers. However, the particular regulation of E2F3 in colon cancer needs further investigation.

**Methods:**

The self-renewal ability was detected through a sphere formation assay. The tumorigenic ability was measured through nude mice *in vivo* assay. The protein expression of genes was examined through a Western blot. The expression of E2F3 in tumor tissues was detected through an IHC assay. The resistance to cisplatin was assessed through the CCK-8 assay. The cell migration and invasion abilities were measured after upregulating or suppressing E2F3 through the Transwell assay.

**Results:**

Results uncovered that E2F3 was upregulated in spheroid cells. In addition, E2F3 facilitates stemness in colon cancer. Moreover, E2F3 facilitated colon cancer cell migration and invasion. Finally, it was revealed that E2F3 affected the STAT3 pathway to modulate stemness in colon cancer. E2F3 served as a promoter regulator in colon cancer, aggravating tumorigenesis and stemness in colon cancer progression through the STAT3 pathway.

**Conclusion:**

E2F3 may be a useful biomarker for anticancer treatment in colon cancer.

## Introduction

Colon cancer is one of the most prevalent tumors and results in cancer-interrelated deaths globally (1, 2). Early surgical operations obviously ameliorate the 5-year survival rate of colon cancer patients, but most advanced colon cancer patients are found to have metastasis and are not suited for surgery (3, 4). Unluckily, treatment options with useful effects for advanced colon cancer patients were few (5, 6). Hence, comprehending the relevant molecular regulatory mechanism of colon cancer progression and looking for accurate and serviceable biomarkers were imperative.

More and more factors (lncRNAs, circRNAs, miRNAs, and proteins) as molecular targets have been disclosed to join in the regulation of cancer’s progression, including colon cancer. For example, lncRNA LINC01578 modulates the NF-κB/YY1 axis to strengthen metastasis in colon cancer (7). CircRNA circCSPP1 combines with miR-431 to regulate ROCK1 and ZEB1, then facilitates the tumorigenesis of colon cancer (8). HOXD13 enhances PTPRN2 expression to aggravate colon cancer progression (9). RNA binding protein PUM1 accelerates tumor growth in colon cancer (10).

E2F transcription factor 3 (E2F3) is one transcription factor that modulates a variety of biological processes, such as DNA synthesis and restoration, centrosome copy, and so on (11, 12). It exhibits dysregulated expression in diversified cancers. For instance, E2F3, modulated by circ-PRKAR1B, facilitates the progression of liver cancer (13). E2F3 aggravates breast cancer tumor metastasis (14). H3K27ac-triggered FOXC2-AS1 enhances E2F3 to strengthen tongue squamous cell carcinoma (15). In addition, miR-34a targets E2F1 and E2F3 to modulate breast cancer tumorigenesis (16). QKI-6 reduces E2F3 to retard the progression of bladder cancer by regulating NF-κB signaling (17). Certainly, E2F3 has also been probed in colon cancer. For example, miR-449b downregulates CCND1 and E2F3 expression to suppress the progression of colon cancer (18). In colon cancer, dysregulation of miR-34a targets Sirt1 and E2F3 to result in drug resistance to 5-FU (19). Apigenin modulates miR-215-5p to reduce E2F1/3 and retards colorectal cancer tumorigenesis (20). However, the regulatory function of E2F3 needs more research in colon cancer.

The signal transducer and activator of the transcription 3 (STAT3) pathway has also been discovered to be a key regulatory pathway that participates in diversified cancers’ progression (21–23). Gene set enrichment analysis has discovered that the critical oncogenic pathway (STAT3) is tightly associated with E2F7/8 (24). In addition, E2F3 and STAT3 are both overexpressed in breast cancer (25). However, the influences of E2F3 on the STAT3 pathway are unknown in colon cancer.

The purpose of our work is to probe the regulatory effects of E2F3 on the tumorigenesis of colon cancer. Our findings demonstrated that E2F3 accelerated stemness in colon cancer by activating the STAT3 pathway. This discovery may provide a valid target for colon cancer’s targeted therapy and mitigate the suffering of colon cancer patients.

## Materials and methods

### Cell line and cell culture

The colon cancer cell line HCT116 was gained from the Cell Bank of the Chinese Academy of Sciences (Shanghai, China) and cultured in RPMI-1640 medium (Gibco, Carlsbad, CA, USA) with 10% fetal bovine serum (FBS), and then maintained at 37°C with 5% CO_2_ in a humidified incubator. Spheroid cells are a small number of tumor cells with stem cell characteristics that are enriched by sphere culture. To verify the function of the STAT3 pathway, colon cancer cells were treated with epidermal growth factor (EGF, 10 ng/ml, Sino Biological, Beijing, China) to stimulate STAT3 activation and selective STAT3 inhibitor S3I-201 (100 μM, MeilunBio, Dalian, China) to suppress STAT3 DNA-binding activity for 24 h.

### Sphere formation assay

RPMI-1640 medium containing 1% antibiotic mixture (100 mg/ml streptomycin and 100 U/ml penicillin G, Gibco), 1% N2 supplement (Invitrogen, Carlsbad, California, USA), 2% B27 supplement (Invitrogen, Carlsbad, California, USA), 20 ng/ml FGF2 (Chemicon, Temecula, CA, USA), and 20 ng/ml EGF (Chemicon, Temecula, CA, USA) was applied to incubate cells in the 24-well plate. After 2 weeks, the spheroids were observed under a microscope (Olympus, Tokyo, Japan) at ×100 magnification.

### *In vivo* assay

Animal studies were conducted in accordance with the institutional guidelines of Beijing Viewsolid Biotechnology Co. Ltd. (Beijing, China). (VS212601450). The male BALB/c nude mice (6 weeks; *n* = 5 in each group) were obtained from Weitong Lihua Experimental Animal Technology (Beijing, China). The 1 × 10^6^ cells were subcutaneously injected into the right flank of nude mice. At 1 month postinjection, the mice were killed, tumors were collected, and pictures of tumors were taken for examining their volumes.

### Western blot

The isolated proteins in RIPA lysis buffer (Beyotime Institute of Biotechnology, Shanghai, China) were electrophoresed with SDS-PAGE followed by moving onto the polyvinylidene fluoride (PVDF) membranes (Amersham Pharmacia Biotech, Sweden). After being blocked, the membrane was probed with primary antibodies including E2F3 (ab152126; dilution 1:500; rabbit; Abcam, Shanghai, China), A2B5 (ab53521; dilution 1 µg/ml; mouse), CD133 (ab222782; dilution 1:2,000; rabbit), Nestin (ab105389; dilution 1:100; rabbit), Sox2 (ab97959; dilution 1 µg/ml; rabbit), and GAPDH (ab8245; dilution 1:500; mouse). Next, HRP-anti-rabbit IgG (ab6721; dilution 1:2,000) was mixed with the membranes. At last, the bands were assessed with the enhanced chemiluminescence system (ECL, Thermo Fisher Scientific Inc, Waltham, MA, USA).

### IHC assay

After being deparaffinized and rehydrated, paraffin sections of tumor tissue from nude mice were mixed with primary anti-E2F3 (ab152126; dilution 1:500; rabbit; Abcam, Shanghai, China) overnight at 4°C. Following washing, appropriate second antibodies (ab6721; dilution 1:1,000; HRP-anti-rabbit) were cultivated with the sections. Next, sections were dyed through 3,3′-diaminobenzidine (DAB) and counterstained with hematoxylin. The images were then captured on a Carl Zeiss LSM880 AiryscanTM confocal microscope (magnification ×100) using ZEN software.

### CCK-8 assay

The cells were grown in 96-well plates and treated with cisplatin (DDP, 40 μM) for 48 h. CCK8 reagent (10 μl, Takara, Dalian, China) was mixed into the well for another 2 h. The cell viability was tested at 450 nm under the Microplate Reader (Bio-Rad, Hercules, California, USA).

### Transwell assay

The Matrigel-coated (Becton Dickinson, Franklin Lakes, NJ, USA) (or not) Transwell chambers (Corning Life Sciences, Corning, NY, USA) were employed for invasion (or migration) assays. The upper chamber was set with cells (1 × 10^4^) and RPMI-1640 medium without FBS, and the lower chamber was set with RPMI-1640 medium with 10% FBS. Next, the invaded or migrated cells were fixed through 90% ethanol and dyed with 0.1% crystal violet. The invaded or migrated cells were examined through the inverted microscope (IX71, magnification ×200, Olympus, Tokyo, Japan). Images were analyzed using ImageJ (National Institutes of Health, NIH, Bethesda, Maryland, USA).

### Statistical analysis

All data were expressed as the mean ± standard deviation (SD). All experiments were conducted in triplicate. SPSS 20.0 software was employed to carry out the statistical analysis. The comparisons were estimated through the Student’s *t*-test (two groups) or one-way ANOVA (multiple groups). *p* < 0.05 was set as a significant difference.

## Results

### E2F3 was upregulated in spheroid cells

**Figure 1:**
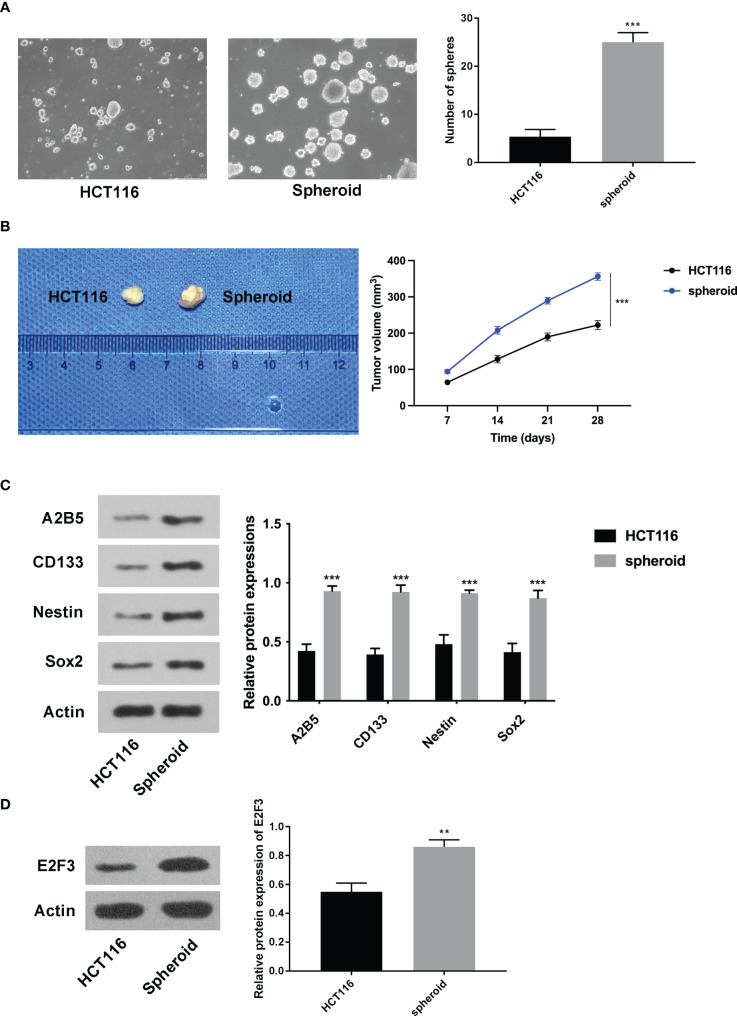
E2F3 was upregulated in spheroid cells. **(A)** The self-renewal ability of spheroid cells and HCT-116 cells was detected through the sphere formation assay. **(B)** The tumorigenic ability of spheroid cells and HCT-116 cells was measured through nude mice *in vivo* assay. **(C)** The protein expression of A2B5, CD133, Nestin, and Sox2 in spheroid cells and HCT-116 cells was assessed through Western blot. **(D)** The expression of E2F3 in spheroid cells and HCT-116 cells was examined through Western blot. ^**^ *p* < 0.01; ^***^ *p* < 0.001.

At first, it was discovered that the increased number of spheres was greater in the spheroid cells than in the HCT116 cells (**Figure 1A**). Furthermore, the tumorigenic ability of spheroid cells was stronger than that of HCT116 cells (**Figure 1B**). In addition, it was discovered that A2B5, CD133, Nestin, and Sox2 were all higher in spheroid cells (**Figure 1C**). Importantly, the expression of E2F3 was upregulated in spheroid cells (**Figure 1D**).

### E2F3 facilitated stemness in colon cancer

**Figure 2:**
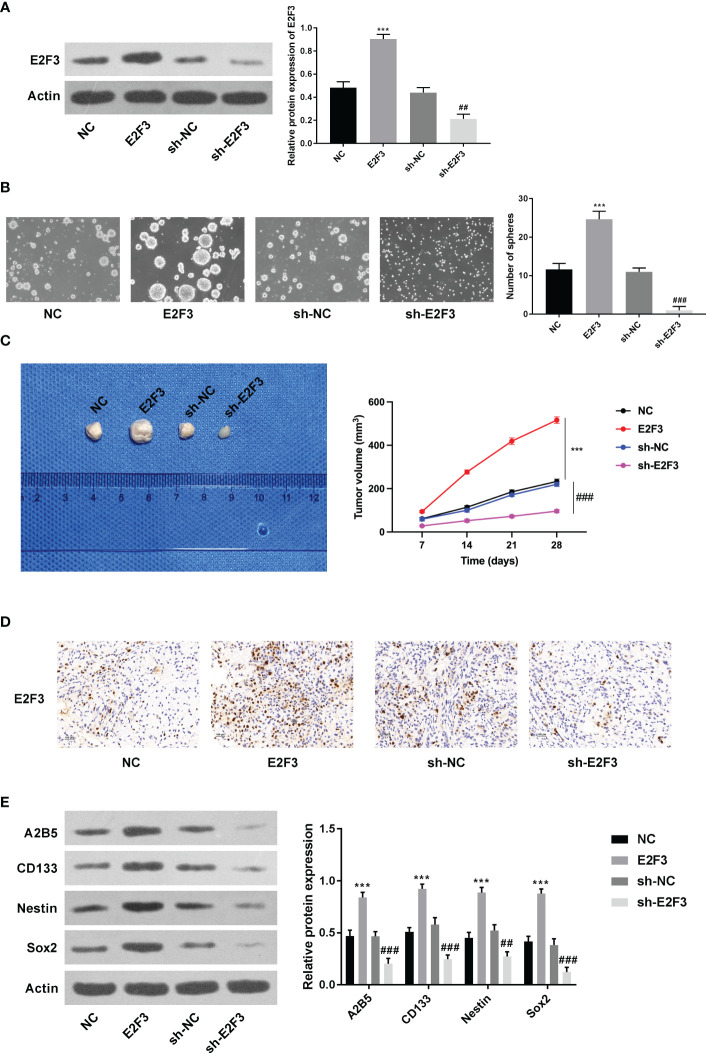
E2F3 facilitates stemness in colon cancer. **(A)** The overexpression and knockdown efficiencies were measured through Western blotting. **(B)** The self-renewal ability was tested after overexpressing or silencing E2F3 through sphere formation assay. Scale bar: 250 μm. **(C)** The tumorigenic ability was examined after up- or downregulating E2F3 through nude mice *in vivo* assay. **(D)** The expression of E2F3 in tumor tissues was detected through an IHC assay. Scale bar: 100 μm. **(E)** The protein expression of A2B5, CD133, Nestin, and Sox2 was inspected through Western blot. ^***^ *p* < 0.001 vs. the NC group; ^##^ *p* < 0.01; ^###^ *p* < 0.001 vs. the sh-NC group.

As displayed in **Figure 2A**, the overexpression and knockdown efficiencies of E2F3 were confirmed. Next, we discovered that the formation ability of spheres was enhanced after E2F3 overexpression and reduced after E2F3 inhibition (**Figure 2B**). Moreover, tumor growth was increased after overexpressing E2F3 and decreased after suppressing E2F3 (**Figure 2C**). Through the IHC assay, E2F3 expression was enhanced after E2F3 overexpression and reduced after E2F3 knockdown (**Figure 2D**). Additionally, a Western blot was performed to measure the stemness-related proteins (A2B5, CD133, Nestin, Sox2) (26, 27). Results from Western blot revealed that the protein levels of A2B5, CD133, Nestin, and Sox2 were upregulated through E2F3 overexpression and downregulated through E2F3 knockdown (**Figure 2E**). Taken together, E2F3 facilitated stemness in colon cancer.

### E2F3 facilitated cell migration and invasion in colon cancer

**Figure 3:**
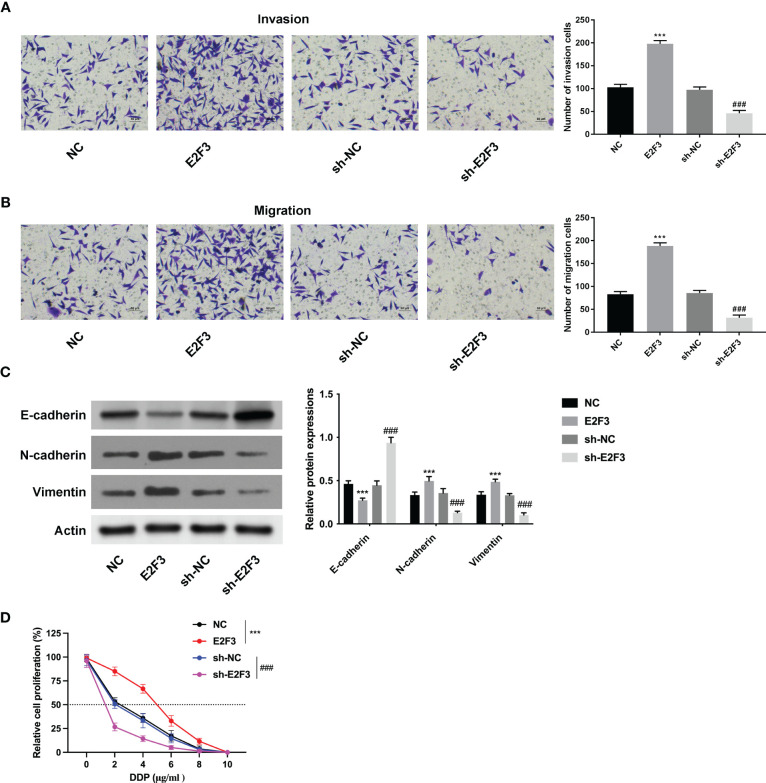
E2F3 facilitates cell migration and invasion in colon cancer. **(A, B)** The cell migration and invasion abilities were measured after upregulating or suppressing E2F3 through Transwell assay. Scale bar: 50 μm. **(C)** The protein expression of E-cadherin, N-cadherin, and vimentin was evaluated after E2F3 overexpression or suppression through Western blot. **(D)** The resistance to cisplatin was assessed after overexpressing or inhibiting E2F3 through CCK-8 assay. ^***^ *p* < 0.001 vs. the NC group; ^###^ *p* < 0.001 vs. the sh-NC group.

Further investigation demonstrated that the migration and invasion abilities were strengthened after upregulating E2F3 and weakened after silencing E2F3 (**Figures 3A, B**). Moreover, the E-cadherin expression was enhanced, and the N-cadherin and vimentin were reduced after E2F3 overexpression. The E-cadherin expression was reduced, and the N-cadherin and vimentin were enhanced after E2F3 inhibition (**Figure 3C**). In addition, the IC_50_ of HCT116 cells transfected with NC, E2F3, sh-NC, or sh-E2F3 plasmids was increased after E2F3 overexpression and decreased after E2F3 repression (**Figure 3D**). These data revealed that E2F3 facilitated cell migration and invasion in colon cancer.

### E2F3 affected the STAT3 pathway in colon cancer

**Figure 4:**
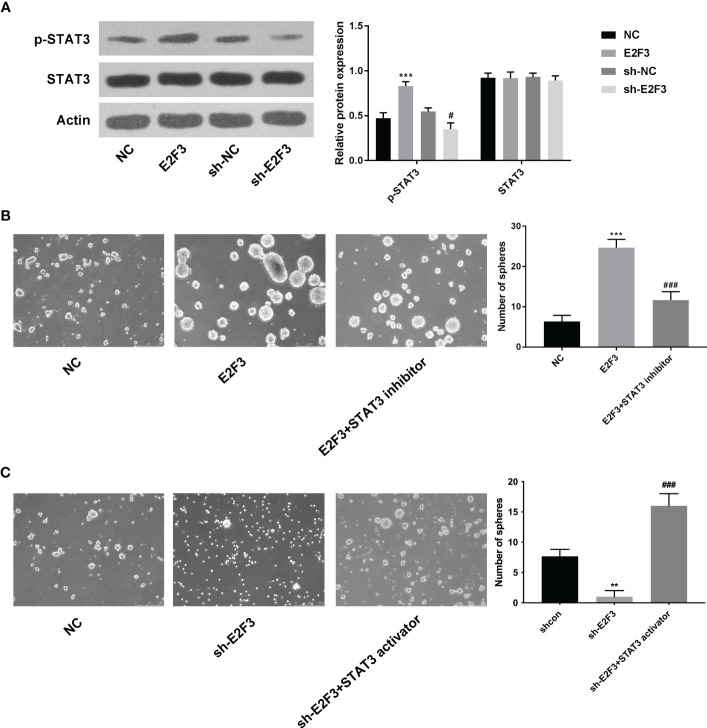
E2F3 affects the STAT3 pathway in colon cancer. **(A)** The p-STAT3 and STAT3 protein expressions were determined after overexpressing or repressing E2F3 through Western blotting. **(B)** The self-renewal ability was tested in the NC, E2F3, and E2F3+STAT3 inhibitor groups through sphere formation assay. Scale bar: 250 μm. **(C)** The self-renewal ability was measured in the shcon, shE2F3, and shE2F3+STAT3 activator groups through a sphere formation assay. Scale bar: 250 μm. ^**^ *p* < 0.01; ^***^ *p* < 0.001 vs. the NC group; ^#^p<0.05 vs the sh-NC group; ^###^ *p* < 0.001 vs. the E2F3 group.

The protein expression of STAT3 was upregulated after overexpressing E2F3 and downregulated after silencing E2F3 (**Figure 4A**). Moreover, the increased spheres mediated by E2F3 overexpression were reversed after STAT3 inhibitor (S3I-201) (**Figure 4B**). Additionally, the decreased spheres mediated by E2F3 knockdown were rescued after the STAT3 activator (STAT3C) (**Figure 4C**). To sum up, E2F3 affected the STAT3 pathway in colon cancer.

## Discussion

E2F3 has been affirmed to be a pivotal facilitator in diverse cancers (13–17), including colon cancer, but its role associated with stemness in colon cancer needs more investigations. In this study, E2F3 expression was upregulated in colon cancer spheroid cells.

Cancer stem cells (CSCs) with invigorative self-renewal and infinite replication capacity are a kind of cell subset that can aggravate the development of malignant tumors (28, 29). CSCs are the major reason for tumor recurrence relapse (30). A growing number of studies are attempted to search for valid bio-targets on stemness in various cancers. For example, CDK1 interacts with Sox2in lung cancer to facilitate stemness (31). Furthermore, HET0016 targets CYP4Z1 in breast cancer to relieve stemness (32). Tanshinone IIA stimulates ferroptosis to suppress stemness in gastric cancer (33). ALG3 modulates the glycosylation of TGF-β receptor II to aggravate stemness and radioresistance in breast cancer (34). Of course, these regulatory effects related to stemness take part in colon cancer. For instance, NF-κB reduces miR-195-5p/497-5p and enhances MCM2 in colon cancer to affect stemness (35). Moreover, miR-139-5p targets E2-2 to attenuate stemness and metastasis in colon cancer (36). In colon cancer, cysteinyl leukotriene receptor 1 contributes to 5-fluorouracil resistance and stemness (37).GATA6 heightens LRH-1 expression in colon cancer to facilitate stemness (38). In this study, we uncovered that E2F3 overexpression facilitated and E2F3 knockdown reduced stemness in colon cancer. Moreover, E2F3 was discovered to aggravate colon cancer cell migration and invasion. Cisplatin is a common drug for colon cancer (39, 40), so a CCK-8 assay was done to investigate cisplatin resistance in colon cancer. It was uncovered that the IC_50_ of DDP was strengthened after E2F3 overexpression and reduced after E2F3 suppression.

The STAT3 pathway is a critical regulatory pathway in diversified cancers, including colon cancer. For instance, HDGF induces the STAT3 signaling pathway in breast cancer to strengthen radioresistance (21). Additionally, NR1D1 retards the JAK/STAT3 signaling pathway to relieve the growth of ovarian cancer (41). Insulin-like growth factor 1 modulates the STAT3 pathway to facilitate cell proliferation and invasion in papillary thyroid cancer (42). P4HB knockdown suppresses the STAT3 signaling to enhance cell apoptosis in colon cancer (43). Resveratrol targets the AKT/STAT3 signaling pathway to relieve colon cancer tumorigenesis (44). Shikonin modulates ADAM17 and the IL-6/STAT3 signaling pathway to attenuate the tumor growth of colon cancer cells and exert synergistic effects by regulating (45). However, the relationship between the STAT3 pathway and E2F3 in colon cancer remains unknown. In the end, it was revealed from this study that E2F3 affected the STAT3 pathway to enhance stemness in colon cancer.

To sum up, it was the first time to uncover that E2F3 acted as a facilitator in colon cancer, strengthening tumor growth and stemness in colon cancer progression by affecting the STAT3 pathway. However, our results regarding the functions of E2F3 in colon cancer remain restrained. In the future, more experiments will be done to further manifest the other regulatory effects of E2F3.

## Data availability statement

The original contributions presented in the study are included in the article/**Supplementary Material**. Further inquiries can be directed to the corresponding author.

## Ethics statement

The animal study was reviewed and approved by Beijing Viewsolid Biotechnology Co. LTD.

## Author contributions

QG and WH designed the study. KA and ZL collected the data. CD and WH analyzed the data. QG wrote the manuscript. All authors contributed to the article and approved the submitted version.
